# Green and Facile Synthesis of Nitrogen and Phosphorus Co-Doped Carbon Quantum Dots towards Fluorescent Ink and Sensing Applications

**DOI:** 10.3390/nano8060386

**Published:** 2018-05-31

**Authors:** Ruiqi Bao, Zhiyi Chen, Zhiwei Zhao, Xuan Sun, Jinyang Zhang, Linrui Hou, Changzhou Yuan

**Affiliations:** 1 School of Material Science and Engineering, University of Jinan, Jinan 250022, China; baorqxq@163.com (R.B.); ah_chenzy@163.com (Z.C.); Azhaozw_ME@163.com (Z.Z.); Asunx_ME@163.com (X.S.); ym0808@sohu.com (J.Z.); 2 School of Materials Science and Engineering, Anhui University of Technology, Ma’anshan 243002, China

**Keywords:** carbon quantum dots, N/P co-doped, tunable photoluminescence, fluorescent ink, fluorescence sensor

## Abstract

Fluorescent carbon quantum dots (CQDs) have held great promise in analytical and environmental fields thanks to their congenitally fascinating virtues. However, low quantum yield (QY) and modest fluorescent stability still restrict their practical applications. In this investigation, a green hydrothermal strategy has been devised to produce water-soluble nitrogen/phosphorus (N/P) co-doped CQDs from edible *Eleocharis dulcis* with multi-heteroatoms. Without any additives and further surface modifications, the resultant CQDs exhibited tunable photoluminescence just by changing hydrothermal temperatures. Appealingly, they showed remarkable excitation-dependent emission, high QY, superior fluorescence stability, and long lifetime. By extending the CQDs solutions as a “fluorescent ink”, we found their potential application in the anti-counterfeit field. When further evaluated as a fluorescence sensor, the N/P co-doped CQDs demonstrated a wide-range determination capability in inorganic cations, and especially the remarkable sensitivity and selectivity for elemental Fe^3+^. More significantly, the green methodology we developed here can be readily generalized for scalable production of high-quality CQDs with tunable emission for versatile applications.

## 1. Introduction

Recently, carbon quantum dots (CQDs) with the size of 2–10 nm have emerged as the promising photoluminescent (PL) material due to their high stability, low toxicity, excellent biocompatibility, versatile surface chemistry, and cost-efficient nature along with easy availability [1,2]. Compared to traditional PL materials (such as organic dyes and semiconductor quantum dots), these attractive features of CQDs inherently make them as the most promising alternatives in a wide range of applications including bioimaging, light emitting diodes (LEDs), sensors, energy-saving display, optoelectronic devices, and so on [1,2,3,4,5,6]. As a result, considerable attentions have been paid to exploring a variety of approaches for fine synthesis of CQDs, such as vigorous chemical oxidation of carbon sources [7], laser ablation [8], microwave-assisted method [9,10], ultrasonic synthesis [11], pyrolysis [12], electrochemical etching [13], and hydrothermal methods [14,15]. Among these synthetic strategies above, the hydrothermal method possesses several distinct superiorities benefiting from its simple, green, low power-consumption, and scalable feature [15]. Additionally, a series of carbon sources, including coal [16], graphite oxide [17], formaldehyde [18], carbon nanotubes [19], carbon soot from natural gas [20], fruit juices [21], peels [22], grass [23], plant leaves [24], and so on, have been widely investigated. In general, a Teflon-lined autoclave over hydrothermal treatment can provide a specific temperature and pressure for the dehydration of the carbon precursors [14,15,18]. With the proceeding of dehydration reaction, CQDs with a tunable degree of carbonization occurs, and these CQDs usually consist of carbon, hydrogen, oxygen, and even other heteroatoms decorated with numerous functional groups on their surface [18,23]. Thus, the mechanism proposed is inclined to be a dehydration process, rendering the formation of CQDs [18]. Additionally, some CQDs with tunable PL and high quantum yield (QY) or emitting at long wavelength have been reported so far [25,26,27]. Impressively, Li and his co-workers prepared the blue-emitting CQDs with a QY as high as 94.5% via a one-step hydrothermal method [27]. In spite of these successful contributions made for controllable fabrication of CQDs, the low-cost and massive production of high-quality CQDs are still highly desirable.

As is known to all, heteroatoms doped carbon nanomaterials always can improve their intrinsic properties and greatly expand their applications in electrochemical, photocatalytic, bioimaging, and sensing applications [28,29,30,31]. As expected, enormous efforts have been extensively devoted to prepare diverse types of heteroatom-doped CQDs with good PL properties [17,21,32,33,34,35]. For example, Lin et al. synthesized N-doped fluorescent CQDs by using a popular antibiotic-aminosalicylic acid as a precursor [35]. Lee and his co-workers reported the facile fabrication of nitrogen-doped CQDs from the C_3_N_4_ towards a fluorescence-based in vitro and in vivo thermometer [36]. Up until now, the doped atoms in fluorescent carbon have been mainly focused on the nitrogen species. Appealingly, other heteroatoms (i.e., S, P) have recently been gradually introduced into CQDs [31,33]. However, the CQDs jointly containing multiple heteroatoms are still actively pursued, as they generally demonstrate much stronger and/or more adjustable PL properties in contrast to simple CQDs [32]. In common, to obtain heteroatom-doped carbon materials, some heteroatom-containing reagents should be additionally introduced into the synthetic procedures for CQDs, which undoubtedly suffers from some apparent drawbacks, including expensive or poisonous precursors, time-consuming procedures and harsh post-treatment conditions [33,34,35,36,37,38]. Therefore, it still remains a challenge to develop an efficient and green strategy for facile fabrication of multi-heteroatoms co-doped CQDs with excellent fluorescent properties on a large scale.

Up to now, metal ion pollution has gradually become a worldwide issue owing to their serious damage to the environment and human health [15,21]. Various CQDs have been exploited as fluorescent nanosensors for the determination of metal ions based on the fluorescence change in aqueous solutions [21,24]. One should note that Fe^3+^ is an indispensable element for living organisms. Nevertheless, the deficiency and overload of Fe^3+^ ion in the human body can induce an acknowledged risk of diseases, including liver injury, heart disorder, cancer, and so on [39,40,41]. Thus, it is of vital importance to sensitively, yet selectively, detect Fe^3+^ ions in biological, medical, and environmental samples. So far a variety of optical sensors, such as functionalized metal-organic frameworks [39], noble metal quantum clusters [40], and dye-based sensors [41], have been applied to detect the Fe^3+^. Unfortunately, these optical probes often suffer from time-consuming synthesis routes, and/or involve toxic or expensive reagents.

Herein, we present a simple, low-cost and green synthetic strategy towards the water-soluble multi-colored nitrogen/phosphorus (N/P) co-doped CQDs via one-step hydrothermal treatment of the *Eleocharis dulcis* juice without any more additive. With fine adjustment in hydrothermal temperatures from 90 °C to 150 °C, the *Eleocharis dulcis*-derived CQDs exhibited tunable fluorescent colors including navy blue, blue, and cyan. Moreover, the as-synthesized N/P co-doped CQDs showed strong fluorescence, which is highly stable not only under a high ionic strength environment, but also under UV light irradiation, treatment with constant temperatures, and various acidic/neutral/alkaline conditions. Owing to their fluorescent nature, the potential use as an invisible fluorescent ink was assessed. When further utilized as a promising nanosensor for ion detection, the resulting N/P co-doped CQDs appealingly exhibited striking ion-determination capability with the highest sensitivity and selectivity for the Fe^3+^ thanks to the smart introduction of N/P heteroatoms.

## 2. Experimental

### 2.1. Chemicals

All the aqueous solutions were prepared with Milli-Q water from a Milli-Q Plus system (Millipore, MA, USA). ZnCl_2_, NiCl_2_, NaCl, MnCl_2_, MgCl_2_, LiCl, KCl, FeCl_3_, CuCl_2_, CrCl_3_, CoCl_2_, CaCl_2_, BaCl_2_, and AlCl_3_ were purchased from Sinopharm Chemical Reagent Co., Ltd. (Shanghai, China). These chemicals were all of analytical grade, and directly used without any further purification. 

### 2.2. Synthesis of N/P Co-Doped CQDs

**Scheme 1:**
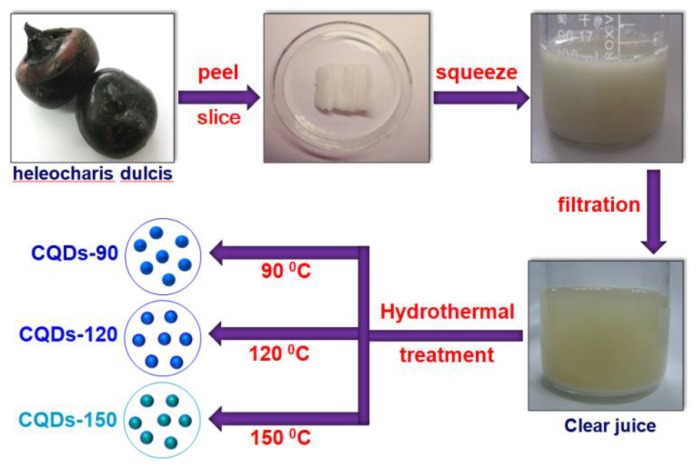
Schematic illustration for facile synthesis of N/P co-doped CQDs from *Eleocharis dulcis*.

N/P co-doped CQDs were synthesized by using *Eleocharis dulcis* as a carbon source through the hydrothermal method at various temperatures, as illustrated in Scheme 1. Fresh *Eleocharis dulcis* was purchased from local supermarket, and washed several times with water. After peeling, the white sarcocarp was chopped, and then squeezed into a juice. After filtration, 40 mL of the obtained clear juice was transferred into a Teflon-lined autoclave (50 mL). After being maintained at 120 °C for 5 h, the autoclave was naturally cooled down to room temperature (RT), yielding a dark brown solution. The solution was then centrifuged at 12,000 rpm for 10 min. Afterwards, the supernatant solution was collected, filtered with a microporous membrane (0.22 µm), and finally subjected to dialysis (1000 MWCO) to eliminate the overreacted residues. The obtained sample hereafter was denoted as the CQDs-120 for convenience. For comparison, a similar synthetic procedure was undertaken to prepare other CQDs just by changing temperature as 90 °C and 150 °C, which were, thus, designed as the CQDs-90 and CQDs-150, respectively.

### 2.3. QY Measurement

The QYs of the as-prepared CQDs were calculated by a relative method with quinine sulfate dissolved in 0.1 M H_2_SO_4_ (QY = 0.546) as the reference [6]. The absorbance below 0.1 was adjusted for the concentration of the samples to minimize the inner filter effect. The QY of CQDs was determined according to the following equation:

$$\mathrm{QY}={\mathrm{QY}}_{r}\cdot \frac{F}{F_{r}}\cdot \frac{A_{r}}{A}\cdot (\frac{n}{n_{r}}{)}^{2}$$

where *F*, *A* and *n* separately present the integrated area of emission, the absorbance at the excited wavelength, and the refractive index for the obtained sample. And the QY*_r_*, *F_r_*, *A_r_*, and *n_r_* are the fluorescence QY, integrated area of emission, the absorbance at the excited wavelength, and the refractive index for the reference, respectively. 

### 2.4. Fluorescence Ink Evaluation

By separately loading the solutions of CQD-90, CQD-120, and CQD-150 into three fountain pens, we wrote the letters of “AHU” on filter paper, and took photos under daylight and UV light (365 nm), respectively. 

### 2.5. Metal Ion Detection

The N/P co-doped CQDs-120 solution (0.01 mg/mL) was used as a model to detect various metal ions (ion concentration: 10^−3^ M) including Zn^2+^, Ni^2+^, Na^+^, Mn^2+^, Mg^2+^, Li^+^, K^+^, Fe^3+^, Cu^2+^, Cr^3+^, Co^2+^, Ca^2+^, Ba^2+^, and Al^3+^. The PL spectra were all recorded after reaction for 5 min at RT. The excited wavelength was set at 380 nm.

### 2.6. Materials Characterizations

The crystallographic phase was examined by powder X-ray diffraction (XRD) (Ultima IV, Rigaku, Japan) using a Cu K*a* source (λ = 0.154056 nm) at a scanning speed of 3° min^−1^ over a 2θ range of 10°–60°. The morphologies of the CQDs were determined by high-resolution transmission electron microscope (HRTEM) (JEOL JEM 2100 system operating at 200 kV, Akishima-shi, Tokyo, Japan). Fourier transform infrared (FT-IR) spectra were recorded on a 360 Nicolet AVATAR FTIR spectrophotometer (Madison, Wisconsin, USA). X-ray photoelectron spectra (XPS) measurement was conducted on a VGESCALAB MKII X-ray photoelectron spectrometer (Cambridge, Cambridgeshire, England) with Mg *ka* excitation source (1253.6 eV). Raman spectra were recorded on a DXR Raman microscope (New York, State of New York, USA). Ultraviolet-visible (UV–VIS) absorption spectra were performed on a Shimadzu UV-3600 UV–VIS spectrometer (Kyoto, Kyoto-fu, Japan). PL spectra and fluorescence decay spectra were obtained by using an Edinburgh FLS980 instrument (Edinburgh, Scotland, England).

## 3. Results and Discussion

In this contribution, low-cost *Eleocharis dulcis*, as a popular edible food, was first used as the sole precursor for the simple fabrication of the N/P co-doped CQDs. As we all know, *Eleocharis dulcis* connately contains carbohydrates, proteins, vitamins (vitamin A, B_1_, B_2_, B_3_, C, E), minerals (Ca, P and Fe) as well as an assortment of phytochemicals (carotenoids), which endows it with abundance in the elemental C, N, O, and P. These unique compositions mean that *Eleocharis dulcis* may be an ideal precursor for fabricating N/P co-doped CQDs. 

**Figure 1:**
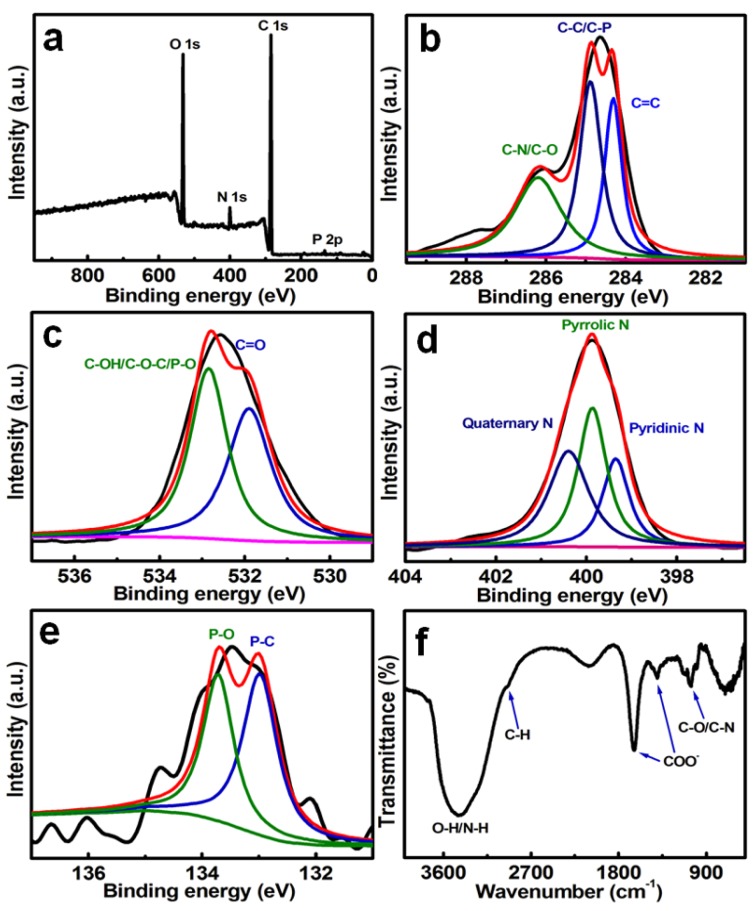
(**a**–**e**) XPS spectra and corresponding fitting profiles (**a**) survey spectrum; (**b**) C 1s; (**c**) O 1s; (**d**) N 1s; (**e**) P 2p; and (**f**) FT-IR spectrum for the CQDs-120.

XPS characterizations were commonly conducted to verify the surface composition. In this connection, the surface composition and element analysis of the as-prepared CQDs were developed via XPS characterizations by using CQDs-120 as a model. Typical XPS data are collectively shown in Figure 1a–e. The overview spectrum (Figure 1a) shows four distinct peaks at ~133.4, ~284.6, ~399.8, and ~532.6 eV, corresponding to P 2p, C 1s, N 1s, and O 1s peaks for the CQDs-120, respectively. The elemental analysis (Table S1, ESI) reveals that the resultant CQDs are composed of elemental C (~72.5 at%), O (~23.6 at%), N (~3.6 at%) and P (~0.3 at%), indicating the successful synthesis of the N/P co-doped CQDs. The C 1s spectrum (Figure 1b) shows four fitted peaks at ~284.3, ~284.9 and ~286.1 eV, which are attributed to sp^2^ C=C, C-C/C-P and C-N/C-O, respectively [42,43,44]. The O 1s spectrum (Figure 1c) is deconvoluted into two peaks at ~531.9 and ~532.9 eV, which can be separately ascribed to C=O and C-OH/C-O-C/P-O functional groups in the CQDs [42,44]. The N 1s spectrum (Figure 1d) demonstrates three N-doping forms including the pyridinic (~399.5 eV), pyrrollic (~399.9 eV) and quandary (~400.4 eV) N atoms. The binding energy peaks for the P 2p (Figure 1e) at ~133.0 and ~133.7 eV confirm the presence of P–C and P–O bonds, respectively. Chemical and structural information about the CQDs-120 was further identified via FT-IR measurement. Figure 1f shows the FT-IR spectrum of the CQDs-120. The peak at around 1057 cm^−1^ is assigned to the vibrations of C-O/C-N bands. And peaks at ~1412 and ~1647 cm^−1^ are ascribed to the COO^−^ group, which should be responsible well for the excellent solubility of CQDs in water. The peak at ~2934 cm^−1^ corresponds to C–H bond, and the broad band at ~3295–~3587 cm^−1^ appears owing to the O–H and N–H bonds. Obviously, these results mean that the *Eleocharis dulcis*-derived products are N/P co-doped carbon materials with high oxygen content, whose surface is decorated hydroxyl, carbonyl/carboxylate groups, ensuring their excellent solubility in water and high stability. The ζ-potential of the CQDs-120 is measured to be −12.5 mV, which can be ascribed to the presence of hydroxyl and carbonyl/carboxylate groups on the CQDs surface. 

**Figure 2:**
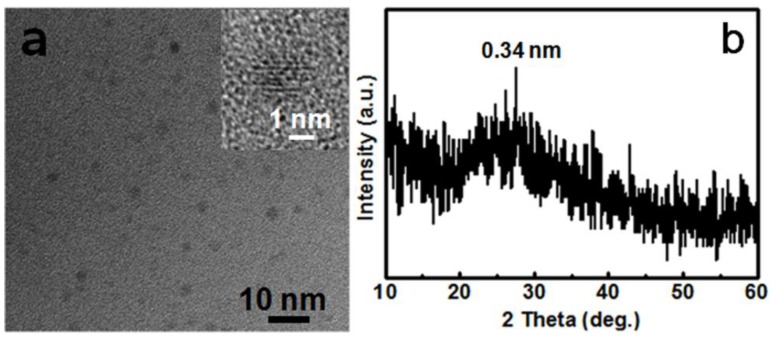
(**a**) TEM image and (**b**) XRD pattern of the CQDs-120.

Figure 2a shows typical TEM image of the CQDs-120. Apparently, the well-dispersed CQDs are typically spherical, and their diameters are mainly located in the range of 2–4 nm. The HRTEM image (the inset in Figure 2a) clearly exhibits the parallel lattice fringe with a spacing of ~0.34 nm, which is in good agreement with the (002) lattice plane of graphitic carbon. The X-ray diffraction is commonly applied to figure out the crystallinity of CQDs. As demonstrated in Figure 2b, a broad diffraction peak centered at ~25° is observed for the CQDs-120, probably due to highly disordered carbon with heteratom doping [45], corresponding to an interlayer spacing of 0.34 nm, which is consistent with the HRTEM analysis above. Additionally, two peaks located at ~1356.5 and ~1594.5 cm^−^^1^ are clearly observed in the Raman spectrum (Figure S1, ESI) of the CQDs-120, typically corresponding to disordered D-band and crystalline G-band, respectively. Additionally, the relative intensity of D-band and G-band is calculated to be around 1.03. 

**Figure 3:**
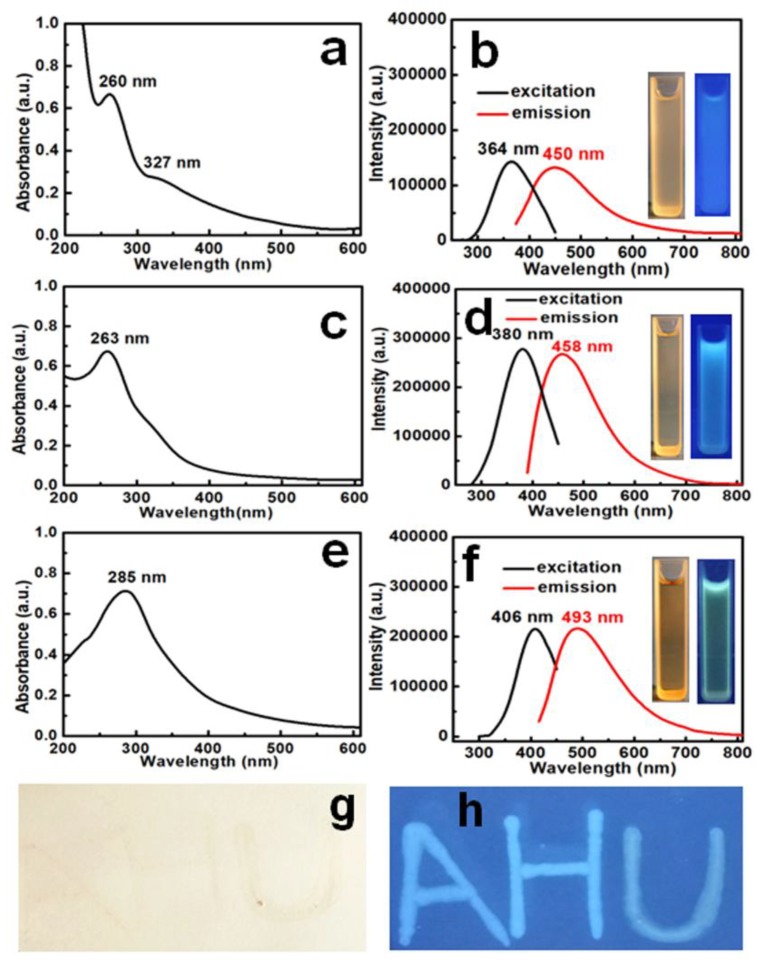
(**a**,**c**,**e**) UV–VIS absorption spectra and (**b**,**d**,**f**) optical excitation and emission spectra of (**a**,**b**) CQDs-90, (**c**,**d**) CQDs-120 and (**e**,**f**) CQDs-150. (**g**,**h**). Digital photographs of the handwritten letters of “AHU” on the filter paper radiated under daylight and UV light (365 nm), respectively. The insets in panels (**b**,**d**,**f**) for the corresponding photographs taken under daylight (**left**) and 365 nm UV light (**right**).

To examine the optical properties of the N/P co-doped CQDs, the original ivory juice squeezing from *Eleocharis dulcis* undergoes firstly UV light irradiation, and it is found that the precursor solution is non-emissive at all upon UV light irradiation. Afterwards, the UV–VIS absorption, optical excitation, and emission spectra of the resultant CQDs solutions are investigated detailedly at RT. Figure 3a shows the UV–VIS absorption spectrum of the CQDs-90. The UV–VIS absorption spectrum exhibits two characteristic absorption peaks at 260 and 327 nm, which are rationally ascribed to the π→π* transition of aromatic C=C domains, and the n→π* transition of conjugated C=O in the CQDs-90 [41,46,47,48]. With increasing hydrothermal temperatures from 90 °C to 150 °C, the latter absorption peak gradually disappears and the whole absorption band is progressively broadened, accompanying with a prominent red-shift from 260 to 285 nm, as illustrated in Figure 3a,c,e, respectively. This typical feature evidently indicates that the absorption properties of the as-prepared N/P co-doped CQDs are affected to some extent by hydrothermal temperatures and the higher temperature always results in the absorption band of CQDs at longer wavelength, which is consistent with the previous report [49]. Likewise, the influences of the hydrothermal temperature upon the PL excitation and emission spectra of CQDs were also studied. As exhibited in Figure 3b, the CQDs-90 presents the optimal excitation and emission wavelengths at 364 and 450 nm, respectively. When the reaction temperature is further enhanced, the homologous red shifts of excitation and emission wavelengths are observed as their absorption spectra. Specifically, the maximum excitation/emission peaks separately centered at 380/458 nm for the CQDs-120 (Figure 3d) and 406/493 nm for the CQDs-150 (Figure 3f). Thus, the fluorescent measurements of CQDs-90, CQDs-120 and CQDs-150 are conducted under the maximum excitation wavelengths of 364, 380, and 406 nm, respectively. The insets show corresponding digital photographs of these CQDs solution under the irradiation of daylight (left) and UV light (right). Obviously, all these CQD solutions are light yellow, transparent, and clear under daylight irradiation. The good dispersion of these as-obtained CQDs in water can be reasonably attributed to their small particle diameter and abundant surface organic groups (carbonyl, carboxylic, and hydroxy) derived from the carbonization of *Eleocharis dulcis* [50]. As a sharp contrast, when excited under UV light (365 nm), they all exhibit strong PL properties, and the emission colors change from navy blue, blue, to cyan with the reaction temperature varying from 90 °C, 120 °C, to 150 °C, which visibly confirms the fluorescence-tunable characteristics of the as-prepared CQDs. Meanwhile, the QY of CQDs is also determined by using quinine sulfate as a reference [6,51,52], and corresponding QY results are comparatively presented (Table S2, ESI). Clearly, the CQDs-120 possesses the highest QY of ~11.2%, which is higher than the reported values for CQDs without element dopant [53,54,55]. Owing to the strong electron-withdrawing abilities of their abundant atoms with N, O, and P, the active sites of the CQDs surface can be effectively passivated. These N/O/P hetero-elements are conducive to the stabilization of excitons, and further alter the whole electronic structures of the CQDs, which is of great benefit to their high recombination yield [56,57,58].

**Figure 4:**
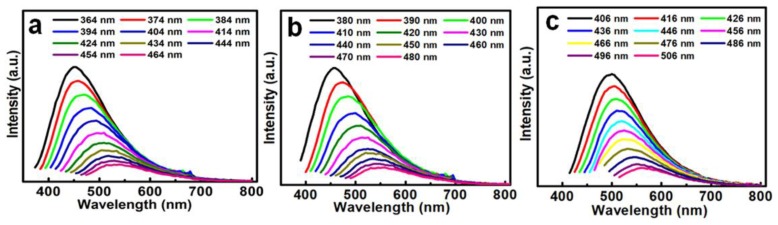
Excitation-dependent PL behaviors of (**a**) CQDs-90, (**b**) CQDs-120, and (**c**) CQDs-150.

To further investigate optical properties of all these CQDs, PL emission spectra were recorded from their strongest excitation wavelengths to the longer wavelengths with 10 nm increments. As displayed in Figure 4a–c, all the CQDs demonstrate similar excitation-dependent PL behaviors, similar to other fluorescent carbon materials reported previously [7,8]. The position of the strongest PL emission peak shifts to longer wavelengths, and PL intensity gradually decreases with the increased excitation wavelength [59,60,61,62]. Such excitation-dependent PL behaviors should be rationally related to the optical selection of differently-sized nanoparticles or distinct surface emission traps in these CQDs or another mechanism altogether [63,64].

**Figure 5:**
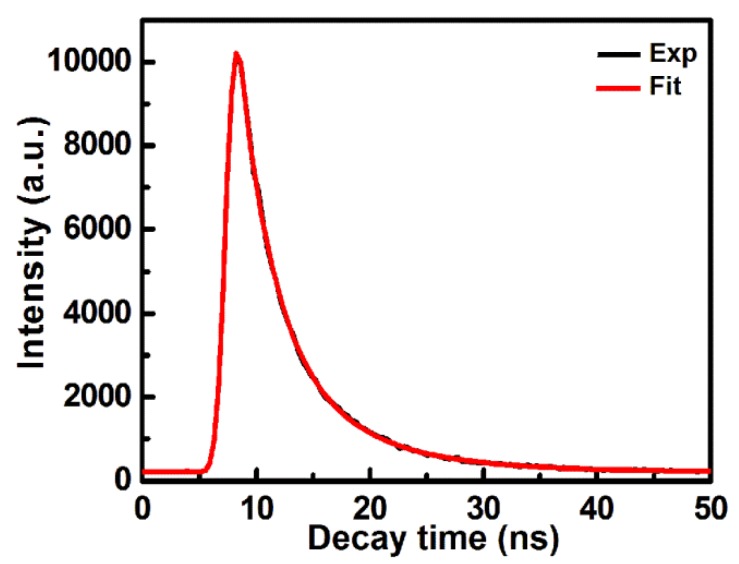
Time-resolved PL spectra of the CQDs-120. The average lifetime of CQDs-120 with two lifetime components (380 nm, decay time at 463 nm emission).

To search thoroughly for the fluorescent mechanism of CQDs, a universal technique of time-correlated single-photon counting (TCSPC) are applied to measure the fluorescent lifetime of CQDs, and the corresponding result for CQD-120 is illustrated in Figure 5. Evidently, the fluorescence decay curves of the CQDs-120 can be fitted well by the following *bi*-exponential formula [65]:

$$Y(x)=A_{1}\exp(-x/t_{1})+A_{2}\exp(-x/t_{2})$$

where *A*_1_ and *A*_2_ are the fractional contributions of time-resolved decay lifetime of *t*_1_ and *t*_2_. The fluorescent progress involves the lifetimes *t*_1_ (2.98 ns) and *t*_2_ (7.80 ns). Specifically, the short lifetime (2.98 ns, ~80.7%) can be related to the intrinsic state of CQDs, and the long lifetime (7.80 ns, ~19.3%) should correspond to the surface state of CQDs [66]. The average lifetime can be calculated as ~3.9 ns, and the short lifetime may basically result from the electron transfer between energy levels of CQDs, which have close relations with the intrinsic state of CQDs. The result implies that the fluorescence in our case is mainly associated with the intrinsic state of CQDs. The fluorescence decay fit well with the best *bi*-exponential function, suggesting that more than one lifetime may be either ascribed to complex energy level, or complex mechanism of fluorescence carbon-based materials [49,67]. Obviously, an ultrafast electron transfer process can be acquired in nanoseconds, which makes the as-prepared CQDs as appropriate candidates for potential applications, such as bioimaging, sensors, and optoelectronic devices.

**Figure 6:**
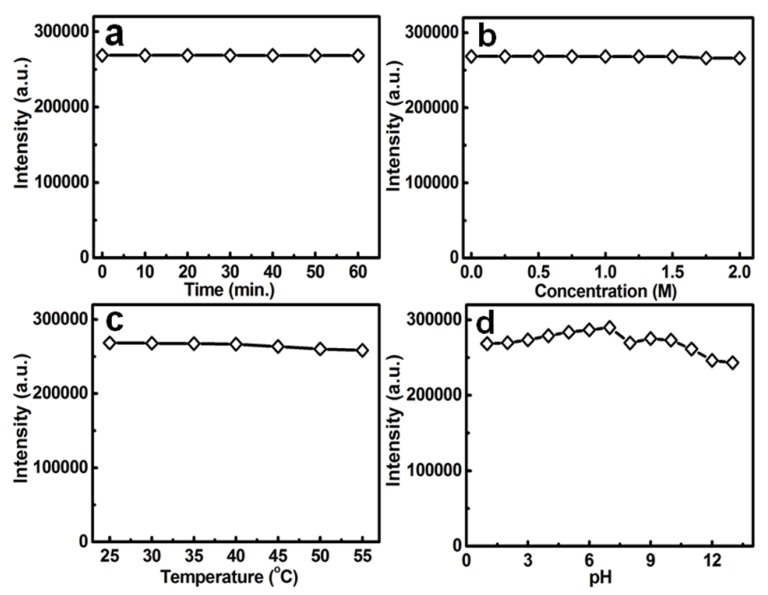
PL intensity variation of the CQDs-120 as a function of (**a**) illumination time of UV lamp with the center wavelength at 365 nm, (**b**) NaCl concentration, (**c**) temperatures, and (**d**) pH values (λ_ex_ = 380 nm).

The stability of CQDs is a vital factor affecting their performance and even practical applications. Accordingly, the PL properties of the CQDs solution under various conditions are investigated in detail by using CQDs-120 sample as a model, as shown in Figure 6. The PL intensity of the CQDs-120 is almost unchanged under continuous irradiation for 60 min (Figure 6a), and even with the NaCl concentration up to a high concentration of 2.0 M (Figure 6b). More strikingly, when the temperature is increased from 25 °C to 55 °C, as plotted in Figure 6c, the PL intensity retention of ~96.5% can still be observed for the CQDs-120. The PL signals of the CQDs-120 at different pH values are also recorded, and typical results are profiled in Figure 6d. Interestingly, the PL intensity shows gradual enhancement with the pH value up to 7, and the maximal response is obtained at pH = 7 accordingly. However, the irregular independence on pH values can be seen when the pH values vary from 7 to 13, which is similar to that reported in the literature [33]. Even so, the PL intensity just changes from 243,239 to 289,836 within the pH range from 1 to 13, which suggests the acceptable stability of the CQDs-120 in acid, neutral, and alkaline solutions to some extent. The comparative discussions above undisputedly confirm the remarkable stability of the CQDs-120, which is of significant importance to its applications.

Owing to the unique luminescence of CQDs, we tentatively apply them as invisible fluorescent ink. By separately loading the solutions of CQD-90, CQD-120, and CQD-150 into three fountain pens, the letters of A, H, and U are handwritten on filter paper. Figure 3g,h present the digital photographs for the handwritten letters of “AHU” radiated under the daylight and UV light (365 nm), respectively. Visually, no luminescence is visible to the naked eye under daylight. Nevertheless, three intense fluorescent colors appear clearly in the writing marks under UV light irradiation. The three-color emitting nature of CQD solutions implies their potential application in anticounterfeit fields, multicolor imaging, and opoelectronic devices. 

**Figure 7:**
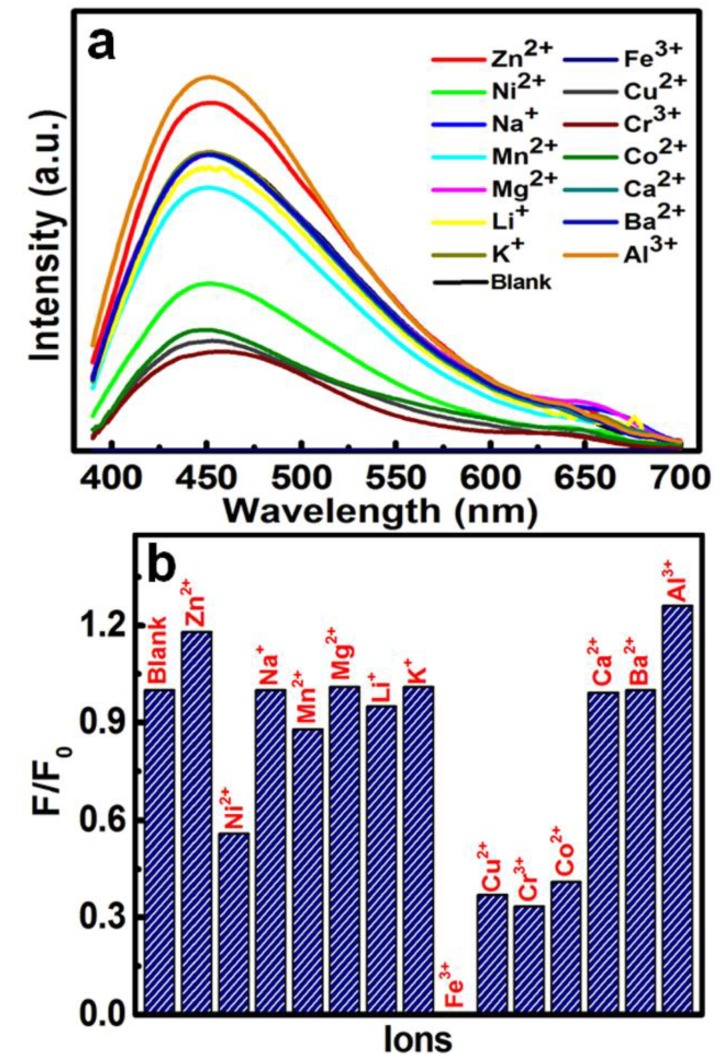
(**a**) Fluorescence responses of the CQDs-120 with different metal ions of 1 mM, and (**b**) corresponding *F*/*F*_0_ with the different metal ions as indicated. *F*_0_ and *F* are the fluorescence intensity before and after addition of the Fe^3+^ ion.

To further expand the application scope of CQDs, we explore the feasibility of the as-prepared CQDs as one fluorescent sensor. In general, the detection and separation of heavy metal ions in water is necessary in business application and/or our daily life, and the fluorescence quenching effect of CQDs has drawn much attention due to its attractive role in ion detection [68,69,70,71]. However, the sensing accuracy [68] and selectivity [69], as well as the range of detection concentrations [70,71] are still needed to be improved. To this end, we examine the PL intensity changes of the CQDs-120 in the presence of representative metal ions (1 mM) under the same condition, such as Zn^2+^, Ni^2+^, Na^+^, Mn^2+^, Mg^2+^, Li^+^, K^+^, Fe^3+^, Cu^2+^, Cr^3+^, Co^2+^, Ca^2+^, Ba^2+^ and Al^3+^, as observed in Figure 7a,b. Clearly, no tremendous effect is observed on PL intensity of CQDs-120 upon addition of Na^+^, Mg^2+^, Li^+^, or K^+^ ions. In contrast, the fluorescence quenching effects are obtained in the presence of representative metal ions, such as Ni^2+^, Mn^2+^, Fe^3+^, Cu^2+^, Cr^3+^, and Co^2+^. Particularly, Fe^3+^ shows the most obvious quenching effect on the PL intensity. The high selectivity of the CQDs-120 for the Fe^3+^ is probably ascribed to the Fe^3+^ with much higher thermodynamic affinity and even faster chelating process toward “N” and “O” of CQDs-120 than other transition-metal ions. Owing to the N/P doping, the formation of coordination bonds between Fe^3+^ and the functional groups on the surface of CQDs become much easier, which is consistent with reported results [72,73,74]. Thus, the radiation transition is disrupted, and the electrons in the excited state of CQDs will transfer to the half-filled 3d orbits of Fe^3+^, inducing nonradiative electron/hole recombination and annihilation, which leads to the fluorescence quenching [74]. 

Conversely, Zn^2+^ leads to the increasing of PL intensity of CQDs, as shown In Figure 7a. The discernable difference can be associated with the different coordination effect between these metal ions and the oxygen-containing functional groups (e.g., –OH and –COOH) on the surface of the CQDs-120 [47,48,49,75]. Convincingly, the results show greater potential application to detect the Fe^3+^ than other metal ions. 

**Figure 8:**
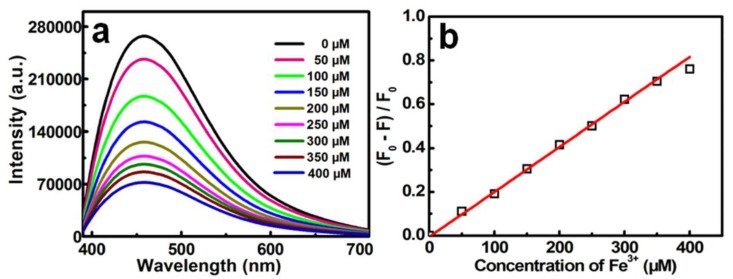
(**a**) PL spectra for the CQDs-120 with various Fe^3+^ concentrations as indicated; and (**b**) the linear relationship of (*F*_0_ − *F*)/*F*_0_ versus the Fe^3+^ concentration.

In spite of elemental iron is an indispensable element in life, the high concentration of the Fe^3+^ is toxic to living organisms, causing various diseases. Therefore, the limit of detection (LOD) of the Fe^3+^ ion is also of vital importance. As a consequence, we examine the PL properties of the CQDs-120 with the addition of different Fe^3+^ concentrations ranging from 0 to 400 μM. As expected, the PL intensity gradually decreases along with the increase in the Fe^3+^ concentration (Figure 8a). In addition to this, the change of the fluorescence intensity ((*F*_0_ − *F*)/*F*_0_) exhibits good linearity with Fe^3+^ concentration in the range of around 50 to 350 μM with a linear equation (*R*^2^ = 0.99033):

$$\frac{F_{0}-F}{F_{0}}=-0.00603+0.00206c$$

where *F*_0_ and *F* indicate the fluorescence intensity at 458 nm in the absence and presence of Fe^3+^ ion, respectively (Figure 8b). The LOD of the CQDs-120 is estimated to be 0.56 μM, which is calculated based on a signal-to-noise of S/N = 3 [76]. The obtained LOD value here is even lower than the limit of standard Fe^3+^ concentration (5.357 μM) for drinking water [74]. It gratifyingly verifies that the CQDs-120 can meet the practical requirement in efficiently sensing the Fe^3+^ ion. Meanwhile, the obtained heteroatoms-enriched CQDs possess both higher QY and better sensitivity towards Fe^3+^ quenching in comparison with the reported CQDs without the element doping [58,59]. The heteroatom-doping effect may contribute to the modulation of the chemical and electronic structure, probably endow them with stronger chelating ability toward Fe^3+^. Moreover, the CQDs presented here demonstrate considerable advantages, of note, which are comparable, and/or even better than, other doped CQDs (Table S3, ESI). The high sensitivity, wide linear range, and excellent selectivity of the as-prepared CQDs-120 for the Fe^3+^ make them an ideal fluorescent probe for real-time tracking of the Fe^3+^ ion. 

## 4. Conclusions

In summary, we presented a simple, yet scalable, synthetic strategy for large-scale production of water-soluble N/P co-doped CQDs from a cheap, green, and readily available natural *Eleocharis* *dulcis* without other chemical additives, which ensures the non-toxicity of the final products. Typically, the *Eleocharis* *dulcis*-derived CQDs exhibited the tunable photoluminescence along with hydrothermal temperatures varying from 90 °C to 150 °C. More impressively, the resultant CQDs demonstrated remarkable excitation-dependent emission, high QY, high fluorescence stability, and long lifetime. The CQDs were utilized as fluorescent ink and sensitive photoluminesence detection for elemental Fe^3+^. The low-cost CQDs displayed their promising application in multi-color imaging and anticounterfeiting fields. Additionally, they also exhibited outstanding selectivity, fast response, and a broad linear detection range from 50 nM to 350 mM. Our investigations here highlight the great potential of the N/P co-doped CQDs in the development of various CQD-based functional materials, anticounterfeiting, and sensing devices.

## Supplementary Materials

The following are available online at http://www.mdpi.com/2079-4991/8/6/386/s1. Quantum yield, elemental compositions, Raman spectrum, and comparisons in the detection of the Fe^3+^ between the CQDs-120 and other CQDs reported in the literature.
