# Supplementary material for: Green and Facile Synthesis of Nitrogen and Phosphorus Co-Doped Carbon Quantum Dots towards Fluorescent Ink and Sensing Applications
Source: Nanomaterials (Basel). 2018 May 31;8(6):386. doi: 10.3390/nano8060386 (PMC6027371; doi:10.3390/nano8060386)
Supplement: Supplementary file 1 [file nanomaterials-08-00386-s001.pdf]

Green and Facile Synthesis of Nitrogen and Phosphorus Co-doped Carbon Quantum Dots towards Fluorescent Ink and Sensing Applications

Ruiqi Bao [2,†], Zhiyi Chen [2,†], Zhiwei Zhao [2], Xuan Sun [1], Jinyang Zhang [1], Linrui Hou [1,*], Changzhou Yuan [1,*]

[1] School of Material Science and Engineering, University of Jinan, Jinan, 250022, P. R. China

[2] School of Materials Science and Engineering, Anhui University of Technology, Ma'anshan, 243002, P.R. China

* Correspondance: houlr629@163.com (Porf. L. R. Hou); mse_yuancz@ujn.edu.cn (Prof. C. Z. Yuan)

† Theses authors contributed equally to this work

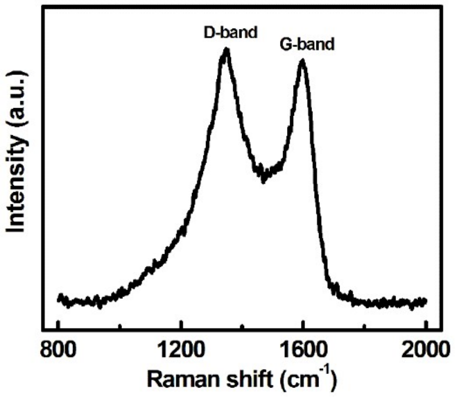

Figure S1 Raman spectrum of the CQDs-120

Table S1 Elemental compositions of the CQDs-120.

Sample	C/at.%	O/at.%	N/at.%	P/at.%
CQDs-120	72.5	23.6	3.6	0.3

Table S2 The quantum yield (QY) of different the N/P co-doped CQDs.

Sample	CQDs-90	CQDs-120	CQDs-150
QY/%	3.3	11.2	8.6

Table S3 Comparisons in the detection of the Fe^{3+} between the CQDs-120 and other CQDs prepared by using different synthetic methods

Method of Synthesis	Material	Linear Range (μM)	LOD (μM)	Ref.
Hydrothermal method	N-doped CQDs	0-2	70	[1]
Hydrothermal method	N-doped CQDs	0-200	0.61	[2]
hydrothermal method	N-doped CQDs	0-50	10.98	[3]
hydrothermal method	N-doped CQDs	0-20	0.32	[4]
hydrothermal method	N-doped CQDs	50-300	10.8	[5]
hydrothermal method	N-doped CQDs	0-50	4.67	[6]
Electrochemical method	graphene CQDs	0-100	7.22	[7]
hydrothermal method	S-doped CQDs	25-250	0.96	[8]
Hydrothermal method	N/S co-doped CQDs	0.002-3	0.22	[9]
Hydrothermal method	N/P co-doped CQDs	1-150	0.33	[10]
Hydrothermal method	N-doped CQDs	2-25	0.9	[11]
Electrochemical method	N-doped CQDs	5-600	1.2	[12]
Hydrothermal method	N/O/P-co-functionalized CQDs	5-350	0.56	Our work

References

[1] R. Atchudan, T. Nesakumar, J. I. Edison, D. Chakradhar, S. Perumal, J. J. Shim, Y. R. Lee, *Sensor. Actuat. B* 2017, 246, 497-509.

[2] Y. B. Wang, Q. Chang and S. L. Hu, *Sensor. Actuat. B*, 2017, 253, 928-933.

[3] Y. Liu, Y. N. Liu, S. J. Park, Y. F. Zhang, T. Kim and S. Chae, *J. Mater. Chem. A*,

2015, 3, 17747-17754.

[4] K. G. Qu, J. S. Wang, J. S. Ren and X. G. Qu, *Chem. Eur. J.*, 2013, 19, 7243-7249.

[5] G. Li, N. Lv, W. Bi, J. Zhang and J. Nia, *New J. Chem.*, 2016, 40, 10213-10218.

[6] J. Yu, C. X. Xu, Z. S. Tian, Y. Lin and Z.L. Shi, *New J. Chem.*, 2016, 40, 2083-2088

[7]A. Ananthanarayanan, X. Wang, P. Routh, B. Sana, S. Lim, D.-H. Kim, K.-H.Lim, J. Li and P. Chen, *Adv. Funct. Mater.*, 2014, 24, 3021-3026.

[8] G. H. Yang, X. J. Wan, Y. K. Su, X. R. Zeng and J. N. Tang, *J. Mater. Chem. A*, 2016, 4, 12841-12849.

[9] Y. F. Chen, Y. Y. Wu, B. Weng, B. Wang and C. M. Li, *Sensor. Actuat. B*, 2016, 223, 689-696

[10] J. F. Shangguan, J. Huang, D. G. He, X. X. He, K. M. Wang, R. Z. Ye, X. Yang, T. P. Qing and J.H. Tang, *Anal. Chem.*, 2017, 89, 7477-7484.

[11] R. Atchudan, T. N. J. I. Edison, K. R. Aseer, S. Perumal, N. Karthik and Y. R. Lee, *Biosens. Bioelectron.*, 2018, 99, 303-311.

[12] F. S. Niu, Y. L. Ying, X. Hua, Y. S. Niu, Y. H. Xu and Y. T. Long, *Carbon*, 2018, 127, 340-348.
